# The impact of the digital economy on Chinese enterprise innovation based on intermediation models with financing constraints

**DOI:** 10.1016/j.heliyon.2023.e13961

**Published:** 2023-02-23

**Authors:** Siying Peng, Xuchu Jiang, Ying Li

**Affiliations:** Zhongnan University of Economics and Law, Wuhan, 430073, China

**Keywords:** Digital economy, Enterprise innovation, Financing constraints, High-quality economic development

## Abstract

The digital economy has become an important driving force for the steady development of China's economy, and enterprise innovation is a key element for an enterprise's survival and development. This paper constructs a mathematical model to measure the scale of digital economic development and the efficiency of enterprise innovation. It builds a fixed effects model and mediated effects model to study the effect of the development of the digital economy on enterprise innovation based on data from 30 provinces from 2012 to 2020. The results show that (1) there is a significant positive effect of the digital economy on enterprise innovation with an impact coefficient of 0.028, and the economic meaning of the coefficient is that for every 1-point increase in the digital economy index, the ratio of R&D capital expenditures to the enterprise's operating income increases by 0.028% points. This finding remains significant in the robustness test. (2) A further test of the mediating effect finds that the digital economy can drive enterprise innovation by reducing financing constraints. (3) In the regional heterogeneity analysis, it is found that the effect of the digital economy in promoting enterprise innovation is more prominent in the central region, and the impact coefficients are 0.04, 0.06, 0.025, and 0.024 for the eastern, central, western, and northeastern regions, respectively. Taking the central region as an example, the economic meaning of the coefficient is that for every 1-point increase in the digital economy index, the ratio of R&D capital expenditures to the enterprise's operating income increases by 0.06% points. The findings of this paper are of practical significance to enterprises in enhancing their innovation capabilities and promoting the high-quality development of China's economy.

## 1 Introduction

Since the reform and opening up, China's economy has made rapid progress. In 2010, China's GDP surpassed that of Japan for the first time, and China became the world's second largest economy. China's economic development has made remarkable achievements. However, most of China's goods are still labor-intensive. China still suffers from various restrictions in many high-tech fields [1]. China urgently needs innovation to add impetus to high-quality economic development. At present, with the continuous improvement of Internet technology, the digital economy has become a new engine for China's high-quality economic development. Compared with the traditional economic form, the digital economy has the characteristics of wide range and fast efficiency. As the foothold of the development of the digital economy, enterprises are also the main body of China's innovation-driven strategy. Research on the digital economy and enterprise innovation is conducive to improving the market competitiveness of micro individuals and to promoting the high-quality development of China's economy.

**Fig. 1:**
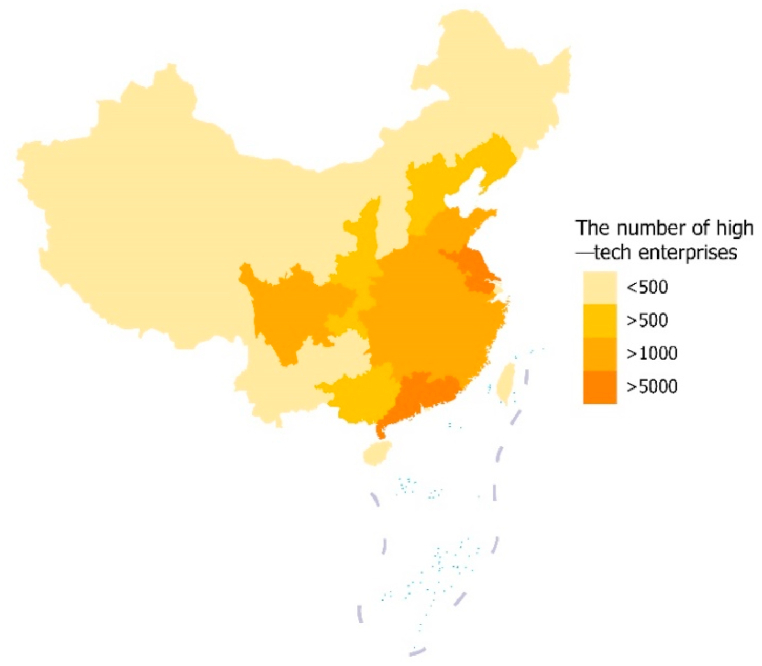
The number of high-tech enterprises in China in 2021.

Zhang et al. [2] proposed that innovation is the primary productive force for enterprises in the face of external competition and internal sustainable development. Improving the level of enterprise innovation is an effective method for enterprises to effectively cope with uncertainty in the business environment, and it is also an important way for China to move toward high-quality and high-level economic development and enhance its international competitiveness. The digital economy is an important direction for future economic growth and is likewise a national strategy. As shown in Fig. 1, there are regional differences in the development of high-tech enterprises in China. The number of high-tech enterprises in the eastern region is greater than that in the western region.

At present, there are many problems in the innovation of Chinese enterprises. Enterprises face problems related to insufficient investment in innovation funds and innovation talent [3]. For the development of an enterprise, capital is often the most important condition and directly determines the success or failure of technological innovation. The prevalence of financial problems in enterprises leads to a lack of motivation to innovate for enterprises. In the process of innovation development, enterprises are more inclined to imitate than to innovate for a lower-cost investment.

The development of the digital economy provides more space for enterprise innovation. On the one hand, the digital economy can help enterprises break the information gap and quickly understand market information by relying on the continuous development of Internet technology. On this basis, the digital economy will help enterprises adjust market strategies in time, increase revenue and reduce costs. More efficient capital operation enables enterprises to provide more funds for innovation. On the other hand, the digital economy can break the financing barriers brought by the traditional financial model to enterprises. The digital economy accelerates enterprises to obtain financial support from the outside, thus providing help for enterprise innovation. Bonina et al. [4] discovered that digital platforms and associated ecosystems provide a promising new environment for enterprise innovation, which is considered a new driving factor for regional innovation efficiency. Wei et al. [5] discovered that enterprise innovation requires a large amount of capital investment and enterprises to recruit high-quality innovative talent. The coexistence of high risk and high profit makes it particularly important for enterprises to make decisions about innovation. Gao et al. [6] found that the problem between the large amount of R&D funding required by enterprises and traditional forms of finance has long been an obstacle to innovation. Williams [7] discovered that the digital economy has solved the financing problem of enterprises with its unique advantages. The digital economy promotes mutual communication between financial institutions and enterprises, helping financial institutions to better serve enterprises. Peng et al. [8] found that on the one hand, for enterprises, the digital economy strategy is the strategic direction for developing modern innovative enterprises. On the other hand, the digital economy promotes the development of enterprise innovation capabilities by breaking down barriers to enterprise financing and solving enterprise funding problems. Currently, there are more diverse dimensions of research on the impact of the digital economy on business innovation. The analysis can be divided into a financial perspective and a policy perspective. From the funding perspective, existing studies have analyzed mainly how easy it is for firms to obtain financial help. From the policy perspective, existing studies have mainly elaborated on the long-term impact of the digital economy on enterprises from the perspective of economic development.

**Fig. 2:**
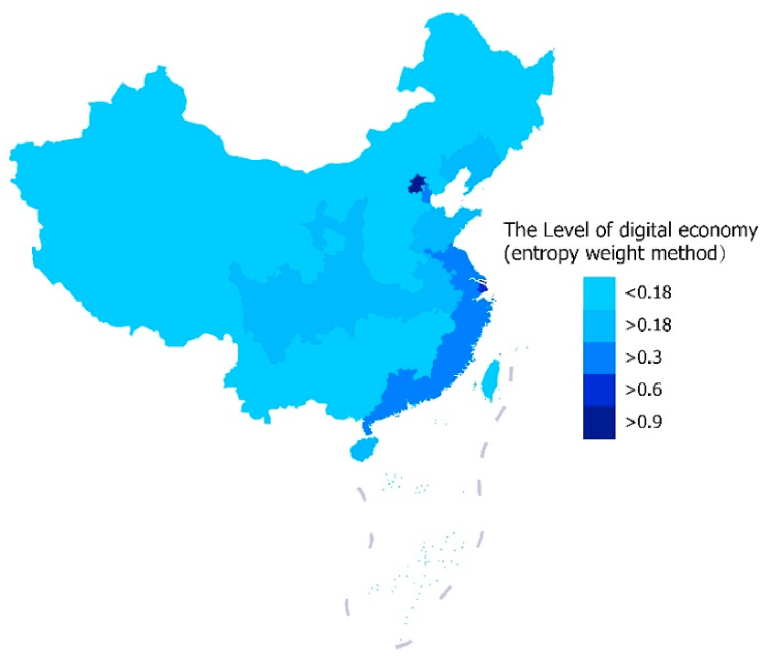
The level of the digital economy in China in 2021.

With the rapid development of Internet technology, economic development has entered the era of the digital economy. The digital economy has garnered substantial attention from scholars in recent years. They mainly analyzed it from a macro perspective. Yang et al. [9] proposed that research on the digital economy should be analyzed from the perspective of financial market risks, Zhao et al. [10] proposed that research on the digital economy should be analyzed from the perspective of high-quality development, and Bulturbayevich et al. [11] proposed that research on the digital economy should be analyzed from the perspective of economic growth. As shown in Fig. 2, the development of the digital economy in China's coastal areas and the capital Beijing is relatively good, while the development of the digital economy in inland areas is lagging.

This paper makes several contributions. First, different from prior research, this study pays attention to the role of the digital economy in enterprise innovation, providing new insight into the study of the digital economy and enriching the research on enterprise innovation. Second, financing constraints are introduced into our study. We identify and examine the intermediary role of financing constraints and clearly show how the digital economy influences enterprise innovation, providing valuable guidelines to help firms comprehend the vital role of financing constraints in promoting enterprise innovation in the digital economy era. Third, we conduct a heterogeneity analysis. We research the impact of the digital economy on firm innovation in four major regions of China and clearly show that the impact of the digital economy on firm innovation differs across regions, providing a theoretical basis and policy support for governments and enterprises.

## 2 Conceptual background

### 2.1 The impact of the digital economy on enterprise innovation

Bai et al. [12] proposed that enterprise innovation capabilities are capabilities that can improve the level of innovation in an enterprise and are usually found in the R&D and marketing aspects of an enterprise. Silva et al. [13] proposed that in terms of research and development, the emergence of the digital economy has accelerated the use of high technology in enterprises, making it possible to extend high technology to the entire industrial chain of enterprises, thus potentially improving their innovation capacity. Nuccio et al. [14] proposed that in the marketing segment, the emergence of the digital economy can better help enterprises match their products with market demand. It not only weakens the traditional market boundaries but also breaks down the market barriers between regions. On the other hand, Zhao [15] proposed that the digital economy improves the efficiency of market resource allocation and increases the degree of market transparency. The increased market transparency intensifies competition among enterprises, so they will take the initiative to innovate production organization methods and business models to maintain their competitive position in the market. In addition, Gaglio et al. [16] proposed that the core underlying technologies of the digital economy lie in artificial intelligence and blockchain technology. The application of these technologies is conducive to the accelerated upgrading of the industry. It gives the digital economy a comparative advantage over the traditional economy in the production, transmission, and utilization of information. The high-speed and efficient transmission and utilization of information promote technological innovation.

First, Xu et al. [17] discovered that production technologies in the context of the digital economy are characterized by low cost and high efficiency, which are conducive to helping enterprises improve marginal returns in the production process. Higher earnings will increase enterprises' willingness to innovate and encourage them to invest more in R&D and engage in innovative activities. Second, the emergence of the digital economy has changed China's financial services model and solved some of the problems that existed under the traditional financial model. Song et al. [18] found that the digital economy effectively reduces the information asymmetry between enterprises and financial institutions by virtue of big data technology, thus reducing the difficulty of financing for enterprises. More efficient financing can help companies find the right balance between production and operation and research and innovation. Finally, Matarazzo et al. [19] proposed that the digital economy has changed the traditional consumption pattern of consumers, which is conducive to enterprises accurately identifying consumer preferences with the help of big data technology to more effectively match between enterprise products and consumer preferences.

At present, research on the relationship between the digital economy and enterprise innovation is more diverse. The core perspectives can be divided into the government perspective, market perspective, and internal perspective. At the level of the government perspective, existing studies mainly analyze from the dimensions of macro policies and government financial investment in R&D. From the market perspective, scholars focus on the impact of market-oriented reforms and industrial agglomeration on the innovation behavior of microentities. At the internal perspective level, scholars mainly focus on the characteristics of enterprise executives, enterprise financial status, and enterprise culture. From a comprehensive perspective, the efficient and sustainable promotion of enterprise innovation is a systemic project. It is determined by a combination of policy, institutional, environmental, and factor supply and demand factors.

Therefore, this paper proposes the following hypotheses to be tested. Research hypothesis $H_{1}$: The digital economy can promote enterprise innovation.

### 2.2 Mechanism for the impact of the digital economy on business innovation

The lower the financing constraints of the enterprise will make the enterprise more liquid. The more conducive this will be to enterprise innovation. Chen et al. [20] proposed that lower financing constraints will make enterprises pay more attention to the renewal and replacement of digital technology. Thus, it is beneficial for enterprises to develop technology platforms to achieve economies of scale. De et al. [21,22] proposed that the lower the degree of financing constraints, the more likely decision makers are to consciously combine data resources with other production factors and skillfully reduce noise and analyze data, making data resources an effective tool for enterprises to gain insight into the market and obtain ideas.

Urbinati et al. [23] proposed that from a cost‒benefit perspective, the higher cost of financing required for innovative activities in the past economy system resulted in the economic benefits generated by an enterprise's innovative activities being much lower than those generated by the original state of business. Therefore, in the traditional economy, companies will forego innovation for the greater good. The development of the digital economy has significantly reduced the costs and increased the benefits of innovation. Mikalef et al. [24] proposed that the gains from innovation will facilitate the enterprise to achieve more capital investment and media attention, which will help to increase the share price and expand production operations, bringing significant economic benefits to the enterprise. In addition, Baldwin et al. [25,26] proposed that due to high financial constraints, enterprises will prefer to pursue tangible assets rather than intangible assets such as technological innovation. This distortion in resource allocation also weakens enterprises' innovation capability and raises the risk of innovation. The digital economy effectively increases the economy's efficiency and availability, mitigates economic imbalances and significantly reduces the risk of business innovation. Reducing innovation risk will make management more willing to make innovation decisions from a long-term perspective. Thus, the impact of the digital economy on the external capabilities of companies and their ability to innovate will significantly contribute to enterprise innovation.

**Fig. 3:**
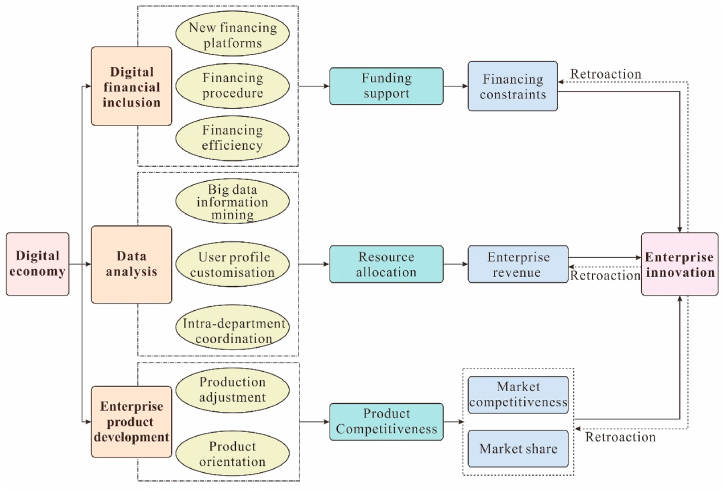
Research framework.

As shown in Fig. 3, we argue that the digital economy can have a catalytic effect on the innovation level of enterprises. Its mechanism is to promote enterprise innovation by easing the financial constraints of enterprises.

Therefore, this paper proposes the following hypotheses to be tested. Research Hypothesis $H_{2}$: The digital economy can promote corporate innovation by easing corporate financing constraints.

## 3 Data sources and model building

### 3.1 Data sources and data processing

The data used in this paper are mainly panel data composed of the digital economy index and financial data of China's Shanghai and Shenzhen A-share listed companies from 2012 to 2020. Among them, the digital economy indicators are derived from the Digital Financial Inclusion Index compiled by the Internet Finance Research Center of Peking University and the China Urban Statistical Yearbook compiled by the National Bureau of Statistics. The digital economy indicators are merged by the city where the enterprise is located to construct panel data on the digital economy and business innovation in prefecture-level cities. The relevant data for listed companies are obtained from the Cathay Pacific database, while for listed companies, this paper aims to process the data according to the following principles: (1) Excluding banking, insurance, finance and special handling (ST) companies during the sample period. (2) We exclude all missing values and outliers from the data of listed companies in the sample period. (3) Reducing the queue of all relevant listed companies' data by 1% tail reduction to eliminate the effect of extreme values. This paper finally obtained 12879 sample data points from 1580 enterprises.

### 3.2 Variable selection

#### 3.2.1 Explained variable

The explained variable of this paper is enterprise innovation. The innovation process consists of two components: input and output. Innovation input includes investment in R&D funds and R&D staff, and innovation output includes patent numbers and the number of patents granted. The long patent grant cycle makes it difficult to collect data, and there are many cases of missing data. Therefore, the number of patent applications is generally used, but the number of patent applications suffers from inaccurate metrics.

The following three dimensions are used to determine the innovation capability of a company: innovation quality, innovation efficiency, and innovation sustainability. The ratio of R&D capital expenditures to the enterprise's operating income is used to measure the firm's innovation capacity mainly from the dimension of innovation efficiency. Innovation efficiency can reflect the input‒output ratio of an enterprise's innovation. When the digital economy increases but innovation output decreases, there is still potential for innovation efficiency to increase if other sources of inputs decline more. Because the indicator takes total firm inputs into account, it is more accurate in exploring the relationship between the digital economy and innovation efficiency.

A better measure is to use R&D capital investment directly to measure autonomous innovation, that is, the ratio of R&D capital expenditure to the company's operating revenue. However, the approach also has the drawback of ignoring the role of innovation outputs. Referring to many studies, this paper finally chooses to use R&D investment to measure the level of independent innovation because R&D funds are more effective and accurate than patent data.

#### 3.2.2 Core explanatory variables

This paper's core explanatory variable is the digital economy's development level. This paper includes the idea of building a digital trading indicator system with the development of the Internet as a core initiative. From the two aspects of Internet development and digital inclusive finance, combined with the availability of relevant data at the city level, this paper comprehensively examines the development level of the digital economy. Huang et al. [27] proposed that the following five indicators can be used to characterize Internet development at the city level: Internet penetration rate, digital workforce base, related outputs, postal service and digital industry application base. The raw data for the above indicators can be obtained from the China Urban Statistical Yearbook. Guo et al. [28] proposed that for the measurement of the development of digital inclusive finance, we can use the China Digital Inclusive Finance Index jointly compiled by the Digital Finance Research Center of Peking University and Ant Financial Services. The data of the above six indicators are processed in the same trend, and the dimension problem is processed. The comprehensive development index of the digital economy is obtained by the entropy weight method. Table 1 shows the construction indicators, indicator attributes and construction process of the comprehensive development level indicator system for each city in China.

**Table 1:** Digital economy index measurement index system.

Level-1 indicator	Level-2 indicator	Level-3 indicator	Weight	Attribute
Digital economy integrated development index	Internet penetration rate	Internet users per 100 people	0.1159	Positive
	Foundation of digital personnel	Computer services and software	0.0935	Positive
	Internet-related output	Total telecom business per capita (Yuan)	0.2385	Positive
	Mail service	Per capita postal service	0.3567	Positive
	Application foundation of the digital industry	Mobile phone users per 100 people	0.0609	Positive
	Inclusive development of digital finance	China digital financial inclusion index	0.1341	Positive

#### 3.2.3 Mediating variables

**Fig. 4:**
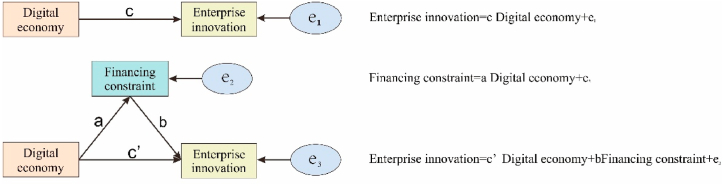
Schematic diagram of the intermediary model.

As shown in Fig. 4, the mediating variables used in this paper are partial mediating variables. The mediating variables affecting firm innovation can be financing constraints, human capital, supply chain concentration, and knowledge networks. The digital economy reduces the information asymmetry between firms and financial institutions and plays a key role in alleviating corporate financing constraints for firms. Therefore, financing constraints are chosen as the mediating variable in this paper. Hadlock et al. [29] proposed using the logarithm of an enterprise's total assets and the enterprise's age to calculate a financing constraint index and use this to measure the degree of financial constraint of the enterprise. The specific method is shown in formula (1). This method is used to measure a firm's financing constraints because these two measures are more exogenous than others. It does not change much with time, and the calculation method is simple. Ariyanto et al. [30] also recognized this calculation method. In this paper, with 1 million yuan as the unit, the calculated SA is negative. The larger the SA index is, the lower the company's financial constraints.

The process is shown in Eq. (1):

(1) $$SA=-0.737Size+0.043{\left(Size\right)}^{2}-0.040Age$$

#### 3.2.4 Control variables

To minimize omitted variable bias, the enterprise size (Size), asset-liability ratio (Lev), return on equity (Roa), equity concentration (Share), total asset turnover (Tat), fixed asset ratio (Ppe), and management rate (Mfee) were selected as the control variables in this paper. To make the regression more objective and scientific, this paper also controls the fixed effects of Year and City.

See Table 2 for all variables, meanings, and sources of data used in this article.

**Table 2:** Definition of primary variables.

Variable classification	Variable name	Variable symbol	Variable definition
Explained variable	Enterprise innovation	Innov	R&D expenditure/total assets
Explanatory variable	Digital economy	Dige	Entropy weight method
Metavariable	Financing constraints	SA	Refer to Eq. (1)
Control variable	Enterprise Scale	Size	Take the log of total assets
	Asset liability ratio	Lev	Total liabilities/total assets
	Return on assets	Roa	Net profit/total assets
	Concentration of ownership	Share	Share proportion of the largest shareholder
	Total Asset turnover	Tat	Sales revenue/total assets
	Fixed assets ratio	Ppe	Fixed assets/total assets
	Management fee rate	Mfee	Overhead/operating income
	Year	Year	Year dummy variable

#### 3.2.5 Model building

Baron [31] proposed that a fixed effects model can be used to test the relationship between the digital economy and enterprise innovation and the mediating effect of financing constraints:

(2) $${Innov}_{it}=a_{0}+a_{1}{Dige}_{it}+\sum a_{k}{Controls}_{it}+\varepsilon_{i}+\mu_{it}$$

(3) $${SA}_{it}=b_{0}+b_{1}{Dige}_{it}+\sum b_{k}{Controls}_{it}+\varepsilon_{i}+\mu_{it}$$

(4) $${Innov}_{it}=c_{0}+c_{1}{Dige}_{it}+c_{2}{SA}_{it}+\sum c_{k}{Controls}_{it}+\varepsilon_{i}+\mu_{it}$$

The fixed effects model is shown in Eq. (2) - Eq. (4), ${Innov}_{it}$ indicates enterprise innovation, ${Dige}_{it}$ indicates the development of the digital economy, ${SA}_{it}$ indicates the level of financing constraints, ${Controls}_{it}$ indicates all the abovementioned control variables, $\varepsilon_{i}$ indicates all unobservable variables that affect Innov but do not change over time and $\mu_{it}$ indicates the random error term.

The above models are all static effects models. Given that the effect of the digital economy on enterprise innovation may be lagged, we select 1 year as the lag period to construct dynamic effects models. Therefore, enterprise innovation is measured at time t, and other variables are measured at time t−1. The dynamic effects models are as follows:

(5) $${Innov}_{it}=a_{0}+a_{1}{Dige}_{it-1}+\sum a_{k}{Controls}_{it-1}+\varepsilon_{i}+\mu_{it-1}$$

(6) $${SA}_{it}=b_{0}+b_{1}{Dige}_{it-1}+\sum b_{k}{Controls}_{it-1}+\varepsilon_{i}+\mu_{it-1}$$

(7) $${Innov}_{it}=c_{0}+c_{1}{Dige}_{it-1}+c_{2}{SA}_{it-1}+\sum c_{k}{Controls}_{it-1}+\varepsilon_{i}+\mu_{it-1}$$

The dynamic effects model is shown in Eq. (5) - Eq. (7), ${Innov}_{it}$ indicates enterprise innovation, ${Dige}_{it}$ indicates the development of the digital economy, ${SA}_{it}$ indicates the level of financing constraints, ${Controls}_{it}$ indicates all the abovementioned control variables, $\varepsilon_{i}$ indicates all unobservable variables that affect Innov but do not change over time and $\mu_{it}$ indicates the random error term.

## 4 Empirical results and analysis

### 4.1 Descriptive statistics

According to the results of the descriptive statistical analysis in Table 3, the mean value of enterprise innovation (Innov) is 0.024, and the maximum value is just 0.097, indicating that most companies invest very little in innovation as a proportion of their business revenue. The digital economy (Dige) has a mean value of 0.047, with a maximum value of 0.422 and a minimum value of 0.006. This indicates that there are some differences in the development of the digital economy in different regions.

**Table 3:** Descriptive analysis of variables.

Variable	Obs	Mean	Std.Dev.	Min	Max
Innov	12879	0.024	0.019	0	0.097
Dige	12879	0.047	0.043	0.006	0.422
SA	12879	−3.265	1.31	−4.831	0
Size	12879	22.109	2.112	0	26.301
Lev	12879	45.654	18.982	0	88.764
Roa	12879	7.585	12.72	−51.579	46.176
Share	12879	32.013	17.183	0	76.44
Tat	12879	0.706	0.415	0	2.387
Ppe	12879	0.21	0.145	0	.644
Mfee	12879	0.085	0.053	0	.282

### 4.2 Correlation analysis

Before regression analysis of the relationship between variables, this paper tests the correlation of variables. The calculation results are shown in Table 4. As seen from the table, the correlation coefficients between the main variables are essentially less than 0.5. In addition, the VIF test for each variable showed that the inflation factor for the variance of the VIF for each variable was less than 2. Therefore, it can be concluded that there is no multicollinearity among the variables of the study model, which qualifies for regression analysis.

**Table 4:** Correlation analysis between the main variables.

Variables	(1)	(2)	(3)	(4)	(5)	(6)	(7)	(8)	(9)	(10)
(1)Innov	1.000									
(2) Dige	0.163	1.000								
(3) SA	0.193	−0.044	1.000							
(4) Size	−0.15	0.066	−0.34	1.000						
(5) Lev	−0.08	0.016	0.023	0.415	1.000					
(6) Roa	0.178	−0.001	0.262	−0.03	−0.12	1.000				
(7) Share	−0.16	−0.002	−0.30	0.308	0.073	−0.035	1.000			
(8) Tat	0.152	0.012	0.133	0.072	0.194	0.286	0.039	1.000		
(9) Ppe	−0.17	−0.153	−0.02	0.160	0.125	−0.113	0.092	−0.042	1.000	
(10) Mfee	0.258	−0.037	0.105	−0.19	−0.30	−0.101	−0.13	−0.409	−0.07	1.000

### 4.3 Empirical analysis

Table 5 presents the results of the benchmark regression of the impact of the digital economy on business innovation. Columns (1) and (2) show the results of the fixed effects and random effects regressions using the full sample, respectively. The Hausman test rejects the original hypothesis (random effects), so we rely mainly on the regression results for fixed effects. Column (2) is a fixed effects regression without the inclusion of mediating variables. The results show that the digital economy has a significant contribution to corporate innovation under the 1% statistical significance test. The coefficient of the core variable Dige is 0.034, which indicates that for every 1-point increase in the digital economy index, the ratio of R&D capital expenditures to the enterprise's operating income increases by 0.034% points. This means that the innovation capacity of enterprises is increasing under the influence of the digital economy, and there is a significant positive relationship between the two. Therefore, these results validate the research hypothesis $H_{1}$: The digital economy can promote enterprise innovation.

**Table 5:** Benchmark regression results.

Variables	(1)Innov	(2)Innov	(3)SA	(4)Innov
Dige	0.034***	0.024***	0.618***	0.028***
	(12.88)	(9.38)	(-11.04)	(10.37)
SA				0.001***
				(9.79)
Size	0.001***	0.001***	−0.169***	0.001***
	(15.68)	(17.95)	(-38.46)	(20.28)
Lev	−0.000***	0.000***	0.151***	0.000***
	(-8.37)	(-5.83)	(23.48)	(-7.82)
Roa	0.000*	0.000***	0.145***	0.000***
	(-1.71)	(-3.42)	(23.60)	(-5.46)
Share	−0.000***	−0.000***	−0.326***	−0.000***
	(-14.61)	(-12.83)	(-54.18)	(-7.07)
Tat	0.013***	0.127***	0.862***	0.012***
	(34.45)	(32.42)	(27.05)	(29.16)
Ppe	0.004***	0.009***	0.881***	0.008***
	(3.63)	(7.58)	(9.52)	(6.72)
Mfee	0.038***	0.024***	5.078***	0.018***
	(16.48)	(10.07)	(26.37)	(7.47)
Year/City	Control	Control	Control	Control
Constant	−0.004**	−0.006***	−0.390***	−0.006***
	(-3.15)	(-6.04)	(0.088)	(-5.66)
Model	Random effects	Fixed effects	Intermediary Model	Fixed effects
Observations	12879	12879	12879	12879
*R*^2^	0.315	0.325	0.398	0.322

Note: *** <0.01, ** <0.05, * <0.1; the number in brackets is the t-stat; the explained variable is enterprise innovation; the core explanatory variable is the digital economy's development level; the estimation method used is fixed effects estimation.

Columns (3) and (4) of Table 5 verify the mediating role of financing constraints. According to the mediation effect test procedure, the first step is to test the relationship between the digital economy and enterprise innovation. The test results in column (2) show that at the 1% significance level, the digital economy's development can significantly enhance an enterprise's independent innovation. The second step is to regress model (2) by adding the financing constraints. The test results in column (3) of Table 5 show that the estimated coefficient of the digital economy is positive at the 1% significance level. The third step is to test whether financing constraints play an intermediary role in the digital economy promoting the independent innovation of enterprises. The test results of columns (2)–(4) of Table 5 show that the estimated coefficients of Dige are all significant at the 1% level, indicating that financing constraints are significant as an intermediary variable. The coefficients of the digital economy and financing constraints are both positive, which shows that the digital economy can alleviate the financing constraints of enterprises and thus promote enterprise innovation. The mediating effect of financing constraints is 2.58% ((0.001*0.618)/0.024). Therefore, these results validate the research hypothesis $H_{2}$: The digital economy can promote corporate innovation by easing corporate financing constraints.

#### 4.3.1 Robustness test

To more accurately portray the impact of the development of the digital economy on enterprise innovation risk and ensure the scientific validity of the findings, this paper will use a robustness test of the benchmark regression by replacing key variables. The entropy weight method is adopted above for the digital economy index measure, and this section uses principal component analysis to rerun the regression above for the digital economy index measure. The regression results are shown in Table 6, which shows that the regression results are still significant after replacing the variables. The financing constraints still play part in the mediating effect, which tests the scientific validity of the findings of this paper. The coefficient of the core variable Dige is 0.001, which indicates that for every 1-point increase in the digital economy index, the ratio of R&D capital expenditures to the enterprise's operating income increases by 0.001% points. The mediating effect value of financing constraints is 1.8% ((0.001*0.018)/0.001). This indicates that the results of this paper are robust and reliable.

**Table 6:** Robustness test of substitution variables.

Variables	(1)Innov	(2)SA	(3)Innov
Dige	0.001***	0.018**	0.001***
	(8.99)	(2.14)	(9.63)
SA			0.001***
			(9.39)
Size	0.001***	−0.232***	0.001***
	(18.53)	(-43.19)	(20.71)
Lev	0.000***	0.017***	0.000***
	(-5.75)	(27.90)	(-7.64)
Roa	0.000*	0.027***	0.000***
	(-3.64)	(33.61)	(-5.63)
Share	−0.000***	−0.015***	0.000***
	(-12.82)	(-24.44)	(-7.28)
Tat	0.012***	0.356***	0.012***
	(32.15)	(13.21)	(28.84)
Ppe	0.008***	0.698***	0.008***
	(7.48)	(9.92)	(6.62)
Mfee	0.022***	3.916***	0.016***
	(9.55)	(18.80)	(6.88)
Year/City	Control	Control	Control
Constant	−0.006**	0.608***	−0.006***
	(-5.91)	(5.39)	(-5.53)
Model	Fixed effects	Intermediary Model	Fixed effects
Observations	12879	12879	12879
*R*^2^	0.315	0.330	0.398

Note: ***<0.01, **<0.05, *<0.1; the number in brackets is the t-stat; the explained variable is enterprise innovation; the core explanatory variable is the digital economy's development level; the estimation method used is fixed effects estimation.

#### 4.3.2 Endogeneity test

The core variables are treated with a first-order lag to solve the endogeneity problem of reverse causality. As shown in Table 7, the sign and significance of the regression coefficients for each variable do not change. The coefficients for the digital economy and financing constraints are both positive, which shows that the digital economy can alleviate the financing constraints of enterprises and thus promote enterprise innovation. The mediating effect value of financing constraints is 2.95% ((0.001*1.241)/0.042). This indicates that the results of this paper are robust and reliable.

**Table 7:** Digital economy lag phase regression results.

Variables	(1)Innov	(2)SA	(3)Innov
L.Dige	0.042***	1.241***	0.032***
	(7.42)	(3.97)	(6.58)
SA			0.001***
			(12.01)
Size	−0.002***	−0.301***	−0.001***
	(-10.66)	(-32.18)	(-9.06)
Lev	−0.000***	0.014***	−0.000***
	(-4.54)	(24.19)	(-6.54)
Roa	0.000***	0.028***	0.000***
	(15.89)	(33.79)	(12.36)
Share	−0.000***	−0.016***	−0.000***
	(-9.18)	(-25.68)	(-6.73)
Tat	0.014***	0.271***	0.013***
	(27.35)	(8.77)	(34.29)
Ppe	−0.006***	0.875***	−0.007***
	(-7.50)	(10.77)	(-7.36)
Mfee	0.238***	2.215***	0.135***
	(34.21)	(9.08)	(19.43)
Year/City	Control	Control	Control
Constant	0.025***	2.015***	0.023***
	(11.45)	(11.29)	(10.42)
Model	Fixed effects	Intermediary Model	Fixed effects
Observations	12879	12879	12879
*R*^2^	0.321	0.357	0.364

Note: ***<0.01, **<0.05, *<0.1; the number in brackets is the t-stat; the explained variable is enterprise innovation; the core explanatory variable is the digital economy's development level; the estimation method used is fixed effects estimation.

#### 4.3.3 Heterogeneity test

Wang et al. [32] propose that since there are large differences among Chinese provinces in various aspects, the digital economy and the development of enterprise innovation show significant heterogeneity in spatial distribution; therefore, it is of practical significance to analyze the impact of the digital economy on enterprise innovation from a regional perspective. For testing purposes, this paper divides China into four regions, namely, the eastern, central, western, and northeastern regions. The regional heterogeneity test results are shown in Table 8. There is a statistically significant positive correlation between the level of digital economy development and the efficiency of enterprise innovation in all four regions. The above results show that the digital economy significantly promotes the development of enterprise innovation in different regions and that the driving effect of the digital economy on the development of enterprise innovation is stronger in the central region than in the eastern region. This is perhaps because the eastern regions have a higher level of digital economy development and a higher level of enterprise innovation because it started earlier than in other regions and is more efficient.

**Table 8:** Heterogeneity test results.

Variables	Eastern Region	Central Region	Western Region	Northeast Region
Dige	0.04***	0.06***	0.025***	0.024***
	(10.50)	(3.49)	(2.05)	(2.50)
Size	−0.001***	0.000*	−0.001***	0.000***
	(-7.80)	(-1.85)	(-3.15)	(-3.46)
Lev	−0.000***	−0.000***	−0.000***	−0.000***
	(-4.31)	(-5.36)	(-5.21)	(-8.31)
Roa	0.000***	0.000***	−0.000	0.000***
	(19.54)	(2.76)	(-0.33)	(13.16)
Share	−0.000***	−0.000***	−0.000***	−0.000***
	(-11.80)	(-4.69)	(-3.89)	(-7.10)
Tat	0.017***	0.017***	0.014***	0.013***
	(38.18)	(16.60)	(9.07)	(32.47)
Ppe	−0.005***	−0.008***	−0.009***	−0.008***
	(-4.48)	(-3.12)	(-2.52)	(-8.97)
Mfee	0.004***	0.126***	0.067***	0.172***
	(48.68)	(15.25)	(5.91)	(39.97)
Year/City	Control	Control	Control	Control
Constant	0.010***	0.013***	0.031***	0.025***
	(5.97)	(3.29)	(4.64)	(3.15)
Model	Fixed effects	Fixed effects	Fixed effects	Fixed effects
Observations	12879	12879	12879	12879
${\;R}^{2}$	0.3173	0.230	0.1871	0.210

Note: ***<0.01, **<0.05, *<0.1; the number in brackets is the t-stat; the explained variable is enterprise innovation; the core explanatory variable is the digital economy's development level; the estimation method used is fixed effects estimation.

## 5 Conclusions

### 5.1 Conclusions

#### 5.1.1 The digital economy can promote enterprise innovation

In the benchmark regression, the coefficient of the core variable Dige is 0.034, which indicates that for every 1-point increase in the digital economy index, the ratio of R&D capital expenditures to the enterprise's operating income increases by 0.034% points. This means that the innovation capacity of enterprises is increasing under the influence of the digital economy, and there is a significant positive relationship between the two. The reason for the positive coefficient is mainly because enterprise innovation activities are often characterized by high risk and long duration, thus requiring significant financial support. Therefore, enterprises are in urgent need of external financial support in the process of innovation. The digital economy has broken the traditional financing barriers and accelerated the speed of enterprise financing, thus effectively improving the innovation capability of enterprises. At the same time, the digital economy helps enterprises market accurately and enhance market competitiveness with technologies such as big data and artificial intelligence, which can help enterprises quickly recover their costs. Therefore, the digital economy can help enterprises to solve their capital needs from internal and external sources, thus promoting their innovation.

The digital economy takes data as the core production factor and relies on artificial intelligence, big data, blockchain, and other relevant digital technologies, which have a significant role in promoting enterprise innovation in theory and practice. The application of the digital economy has an incentive effect on the innovation vitality of enterprises, and there is a significant positive correlation between them. The digital economy brings opportunities to enterprises, stimulates the vitality of economic growth, and enhances market competitiveness.

#### 5.1.2 Financing constraints restrict the innovation and development of enterprises

For the development of enterprises, capital is often the most essential condition and directly determines the success or failure of technological innovation. Enterprises generally have related capital problems and turnover difficulties. The previous financial system is unable to meet the needs of the development and innovation of the companies concerned. The pressure of market competition forces enterprises not only to speed up the operation of funds from inside but also to obtain funds from outside. In this case, the digital economy, with its wide range and high efficiency, has accelerated the speed of enterprises obtaining funds from the outside. More funds can not only help enterprises adjust their strategies flexibly but also help enterprises have more funds for innovation.

#### 5.1.3 The digital economy promotes innovation by reducing financing constraints

The innovation scale of enterprises is highly susceptible to the impact of the company's capital chain. In today's downward pressure on the economy, managers prefer to use the funds available to them for short-term maintenance and growth. Therefore, companies will not shift their focus toward innovative development and long-term growth. In this context, the digital economy effectively reduces the cost of financing for enterprises and effectively reduces the information asymmetry between the two parties in the financial market, thus promoting the willingness and effectiveness of enterprises to innovate.

### 5.2 Policy suggestion

First, it strongly supports the research and development of artificial intelligence, big data and other core technologies of digital economy innovation. Second, improve our financial system and related financial infrastructure. A good financial infrastructure is the basis for reducing enterprises’ financing costs. Gradually improving the financial infrastructure in remote areas is the first step to solve the problems of underdevelopment, obvious geographical disparity, and lack of extensive coverage.

There are differences in digital economic endowment and property rights protection systems in different regions. The promotion of the digital economy should vary according to the actual situation in different regions. For regions with relatively developed Internet technology, we should give full play to the positive role of the digital economy in enterprise innovation and strengthen the enterprise innovation model through digital technology. For regions where the development of the digital economy is relatively slow, we should fully coordinate the development of the digital economy in combination with our own advantages. This type of region should focus on the dividend period of the development of the digital economy on the premise of ensuring that its own development is not too restricted.

### 5.3 Limitations

This paper does not go far enough in analyzing the heterogeneity of the digital economy on enterprise innovation. This paper only analyzes regional heterogeneity for China. However, heterogeneity can be further analyzed in depth from both micro and macro perspectives. Analyzed at the micro level, the impact of the digital economy is different for different types of enterprises. For example, the sensitivity of technology-intensive and labor-intensive firms to the growth of the digital economy is significantly different. First, because technology-intensive companies have more R&D needs and incentives to innovate, they are more sensitive to the growth of the digital economy. Second, because of the rising labor costs in China in recent years, companies are forced to innovate to reduce costs. Analyzed at the macro level, the region's innate resources and institutional protections can also produce heterogeneity. The digital economy endowment can vary from region to region, which leads to differences in the impact of the development of the digital economy on business innovation. Differences in the protection of property rights systems can also arise in different regions. Therefore, a more in-depth study can analyze the heterogeneity at both the micro and macro levels.

This paper focuses on the impact of the digital economy on corporate innovation from a single dimension, i.e., the impact of the digital economy on corporate R&D investment. However, it is also possible to focus on the impact of the digital economy on enterprise innovation from multiple dimensions. For example, it can be analyzed from two dimensions: innovation output and innovation efficiency. This paper mainly examines the impact of the digital economy on corporate innovation from the internal perspective of enterprises. However, the impact of the enterprise's external environment on enterprise innovation is also worth analyzing. Therefore, subsequent studies can further explore the impact of the digital economy on the innovation output and innovation efficiency of firms based on the external environment perspective of enterprises.

## Author contributions

Conceptualization, S.P. ; methodology, X.J. ; formal analysis, S.P. ; data curation, X.J. ; supervision, Y.L. ; writing—original draft preparation, S.P. ; writing—review and editing, X.J. All authors have read and agreed to the published version of the manuscript.

## Funding

The research is supported by Hubei Province Education Science Planning Project (2021GB010), and by Philosophy and Social Sciences Research Project of Hubei Provincial Department of Education (21G032). The authors are grateful to other participants of the project for their cooperation.

## Data availability statement

Data associated with this study has been deposited at:

https://idf.pku.edu.cn/zsbz/index.htm
https://www.stats.gov.cn/
https://www.gtarsc.com/

## Conflicts of interest

The authors declare no conflicts of interest.
